# Analysis of in situ diversity and population structure in Ethiopian cultivated Sorghum bicolor (L.) landraces using phenotypic traits and SSR markers

**DOI:** 10.1186/2193-1801-3-212

**Published:** 2014-04-30

**Authors:** Asfaw Adugna

**Affiliations:** Melkassa Agricultural Research Center, P.O. Box 1085, Adama, Ethiopia

**Keywords:** Diversity, gene flow, phenotypic traits, simple sequence repeat markers, *Sorghum bicolor*

## Abstract

Genetic diversity is a fundamental input for every plant breeding program, genetic resources conservation, and evolutionary studies. *In situ* diversity and population genetic structure of eight cultivated sorghum landrace populations were investigated in the center of origin, Ethiopia using seven phenotypic traits and 12 highly polymorphic sorghum SSR markers. In farmers’ fields, DNA samples were collected using Whatman® plant saver card and quantitative phenotypic traits were measured from 160 individual plant samples belonging to the eight populations representing three diverse geographical regions. High diversity was observed among the various populations for the measured phenotypic traits. The 12 SSR loci produced a total of 123 alleles of which 78 (63.41%) were rare (frequency ≤0.05) with an average of 10.25 alleles per polymorphic locus. The polymorphism information content (PIC) was in the range 0.39-0.85 showing the good discriminatory power of the SSR loci used. Average observed heterozygosity and gene diversity across all populations and loci ranged 0.04-0.33 and 0.41-0.87, respectively. Neighbor-joining and STRUCTURE analyses grouped the 160 samples from the eight populations differently. AMOVA showed 54.44% of the variation to be within populations, 32.76% among populations within regions, and 12.8% among the regions of origin. There was high divergence in the total populations (F_ST_ = 0.40) indicating low level of gene flow (N_m_ = 0.38), but high gene flow was also observed in some adjacent populations. The populations from Wello displayed close relationship with remote Gibe and Metekel populations indicating that the variation followed human migration patterns. Implications of the results for sorghum improvement and germplasm conservation are discussed.

**Electronic supplementary material:**

The online version of this article (doi:10.1186/2193-1801-3-212) contains supplementary material, which is available to authorized users.

## Background

In terms of cultivated area and total grain production, sorghum (*Sorghum bicolor*) is the fifth-most important cereal in the world. It serves as a staple for millions of people in Africa and Asia (Ejeta and Grenier 2005). Africa has become the leading sorghum producer in recent years with an average annual volume contribution of >25 million tons of grain and the area covered by the crop in this continent is larger than in other continents (FAOSTAT 2010). Ethiopia is the third largest producer of sorghum in Africa behind Nigeria and Sudan with a contribution of about 12% of annual production (Wani et al. 2011) and the second after Sudan in the Common Market for Eastern and Southern Africa (COMESA) member countries (USAID 2010). Next to maize and tef, sorghum is the third-most important cereal in Ethiopia (CSA 2012). In Ethiopia, sorghum covers 16% of the total area allocated to grains (cereals, pulses, and oil crops) and 20% of the area covered by cereals (CSA Central Statistical Agency 2011). In 2012 alone, 5.2 million holders produced 3.9 million tons of sorghum grain on 1.9 million hectares of land. More than 95% of this area was covered by landraces. Sorghum is the second most important crop for *injera* (common leavened flat bread) next to tef (Adugna 2012). The grain is used for the preparation of traditional foods, distilled and undistilled beverages and the biomass is highly valued for construction, fuel and animal feed. The crop grows almost exclusively during the main rainy season, which in most regions extends from March to November/December.

Ethiopia serves as the global reservoir for sources of favorable genes of various crops to which it is the Vavilonian center of origin and diversity including sorghum [*Sorghum bicolor* (L.) Moench]. Ethiopian farmers grow mixed sorghum landraces of diverse forms in their fields for various local purposes. The Ethiopian sorghum germplasm has been highly contributing to the global agriculture. Singh and Axtell (1973) identified two high-lysine Ethiopian sorghum lines, IS11167 and IS11758. IS 12662C (SC 171), the source of A2 cytoplasm (the sterile line) for the development of hybrids, which belongs to the Caudatum Nigricans group (Guinea race) was also obtained from Ethiopia (Schertz 1977). Moreover, studies identified two sorghum lines native to Ethiopia (B35 and E36-1) as sources of “stay-green” for drought tolerance, which are currently used in marker assisted breeding programs (Rosenow et al. 1983; Reddy et al. 2009). Wu et al. (2006) identified seven sorghum lines of Ethiopian origin to be resistant to Greenbug biotype I. These are: ETS2140(PI452752), ETS3447(PI455203), ETS3805(PI455812), ETS4159(PI456490), ETS4167(PI456504), ETS4565(PI457212), ETS4614-B(PI457314). Another example is E 35–1, a selection from the Ethiopian zera-zera sorghum landrace, which has now been introduced for direct cultivation and in the breeding programmes in many countries (IBC Institute of Biodiversity Conservation 2007). Moreover, some superior varieties of Ethiopian origin were released in India, Eritrea, Burkina Faso, Zambia, Burundi and Tanzania (Reddy et al. 2006) showing their contribution to the economy of these countries. Being the center of origin and diversity for sorghum, therefore, Ethiopia may harbor unique germplasm that is worthy of crop improvement and conservation.

The significance of studying the genetic diversity of plants is explained elsewhere (e.g., Mutegi et al. 2011). Over the years, a number of studies have been dealt with estimating genetic diversity in cultivated sorghum using phenotypic traits (e.g., Zongo et al. 1993; Appa et al. 1996; Ayana and Bekele 1998; Ayana et al. 2000; Dahlberg et al. 2002; Shehzad et al. 2009), Allozymes (Aldrich et al. 1992; Ayana et al. 2001), RAPDs (Menkir et al. 1997; Ayana et al. 2000; Agrama and Tuinstra 2003; Nkongolo and Nsapato 2003; Uptmoor et al. 2003), RFLPs (Cui et al. 1995; Yang et al. 1996; Jordan et al. 1998), ISSRs (Yang et al. 1996); AFLPs (Uptmoor et al. 2003; Menz et al. 2004; Geleta et al. 2006; Ritter et al. 2007), Genomic SSR markers (Dean et al. 1999; Ghebru et al. 2002; Agrama and Tuinstra 2003; Menz et al. 2004; Casa et al. 2005; Geleta et al. 2006; Barnaud et al. 2007; Deu et al. 2008; Wang et al. 2009; Mutegi et al. 2011; Cuevas and Prom 2013); EST-SSR markers (Ramu et al. 2013); Diversity Arrays Technology (DArT™) (Mace et al. 2008) and SNP markers (Wang et al. 2013; Morris et al. 2013). Some of these studies were based on global and local accessions from gene banks, while others were based on field collections and most of them reported moderate to high diversity. It should be noted that each of these markers has its own advantages and limitations. Moreover, some studies (e.g., Labeyrie et al. 2014) dealt with the influence of ethnolinguistic and cultural diversity on the patterns of genetic structure of sorghum populations.

Phenotypic traits may not give reliable estimate of genetic diversity as these traits are limited in number and due to environmental influence (van Beuningen and Busch 1997). On the contrary, molecular diversity data can potentially bridge conservation and use when employed as a tool for mining germplasm collections for genomic regions associated with adaptive or agronomically important traits (Casa et al. 2005). Simple sequence repeat (SSR) markers are among the markers of choice currently being used for population genetic studies due to their high polymorphism even between closely related individuals within a species (Edwards et al. 1996), transferable between populations (Taramino and Tingey 1996; Gupta et al. 1999), require small amount of DNA, high reproducibility, codominance, abundance, and fairly evenly distributed throughout the euchromatic region of the genome (e.g., Schlotterer 2004). Information on *in situ* diversity and genetic structure of cultivated sorghum using reliable marker systems such as SSRs while indispensible is lacking in the center of origin, Ethiopia. Thus, this study was designed to fill up this gap. Therefore, this study aimed at 1) investigating the genetic diversity of sorghum landraces sampled *in situ* from three agroclimatic regions of Ethiopia using phenotypic traits and SSR markers; 2) investigating the factors shaping the population genetic structure of sorghum landraces; and 3) suggesting measures to aid efforts of crop improvement and genetic resources conservation.

## Materials and methods

### Study areas and plant samples

**Figure 1:**
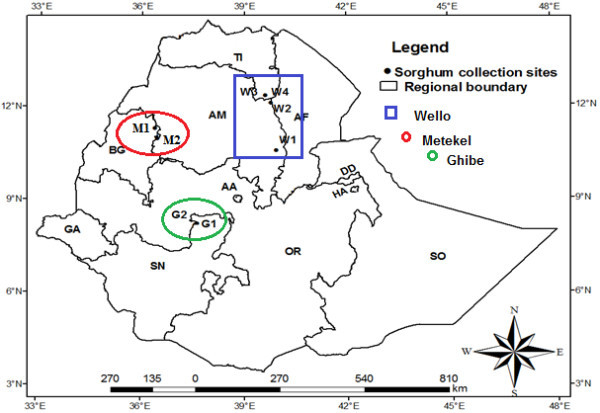
**Map of Ethiopia showing sorghum landrace collection sites.** Key of abbreviations of the regional states: AA = Addis Ababa, AF = Afar, AM = Amhara, BG = Benishangul-Gumuz, DD = Dire Dawa GA = Gambella, HA = Harari, OR = Oromiya, SN = Southern Nations, SO = Somali, and TI = Tigray.

The geographical characteristics of the sample collection sites are presented in Table 1. One-hundred sixty plant samples of cultivated sorghum were collected from eight populations in three diverse geographical and agro-climatic regions of Ethiopia in October and November in 2009 to study *in situ* genetic diversity and population structure. Four of the eight populations were collected in Wello in Amhara regional state (one from south and 3 from north administrative zones), two in Gibe river valley (Oromia regional state), and two in Metekel zone (in Benishangul-Gumuz regional state). The different landrace collections are known by different local names (Table 1). These regions were selected based on four vital reasons: 1) the sites were selected to include a broad swath of the range of sorghum cultivation in Ethiopia; 2) Sorghum is the dominant crop in these regions; 3) Wello region has been under recurrent drought and improved varieties have been introduced into the region as food and seed aid thereby inflicting risk of displacement of the landraces; 4) Metekel and Gibe are high rainfall (>1000 mm) and fertile settlement areas, mainly for people from Wello region due to which sorghum landraces might be displaced by landraces from other regions (e.g., Wello) and other crop species. On the other hand the Wello regions of collection are characterized by low annual rainfall (600-700 mm). All of the regions of collection have high temperatures. In all the regions of collection, long-cycle sorghum landraces are traditionally sown in March/April for the main rainy season and harvested in November/December. Each site of collection was considered to represent a population, from which 20 plants were sampled. Each population was a mixture of different landraces collected from three to five farmers’ fields. The names of the dominant landraces based on farmers’ assignment in each site are presented in Table 1. Readings of the coordinates and altitudes of the collection sites were recorded by a GPSmap *60CSx* Global Positioning System (GPS) (Garmin), which was later overlaid on to the regional map of Ethiopia using ArcGIS version 9.3 (Figure 1).

**Table 1:** **Geographical characteristics of the sorghum collection sites and names of the dominant cultivars**

Geographical zone	Name of specific location	Codes of the collection sites*	Longitude (E)	Latitude (N)	Altitude (m)	Name of dominant cultivar
Gibe	Gibe river bridge side	Gibe-1(G1)	8° 13′	37° 34′	1115	Dalecho
	Gibe-ILRI	Gibe-2(G2)	8° 14′	37° 34′	1149	Key Mashila
Metekel	Pawe settlement village-6	Metekel-1(M1)	11° 18′	36° 24′	1088	Bobe
	Mandura	Metekel-2(M2)	11° 05′	36° 25′	1404	Bobe/Mera mixed
South Wello	Jara Kechema	Wello-1(W1)	10° 30′	39° 56′	1433	Mera
North Wello	Abuare	Wello-2(W2)	12° 05′	39° 39′	1426	76 T_1_#23(improved)
	Kobo town side	Wello-3(W3)	12° 08′	39° 37′	1500	Jigurte
	Alamata-Gerjele	Wello-4(W4)	12° 26′	39° 36′	1486	Degalit

*Letters and figures in parentheses are codes of the collection sites on the Ethiopian map (Figure 1).

### DNA extraction, PCR amplification and genotyping

Leaf squashes were collected *in situ* from mature plants using Whatman® FTA® plant saver card. Extraction and purification of DNA samples were performed using a two-step protocol developed by the manufacturer and optimized for sorghum by Adugna et al. (2011). DNA extraction and the subsequent molecular marker analysis were carried out at Stanley J. Aronoff Laboratory, Ohio State University, Columbus, Ohio. PCR were run using 12 sorghum microsatellite loci that were previously mapped (Brown et al. 1996; Taramino et al. 1997; Bhattramakki et al. 2000; Li et al. 2009) and represented all of the 10 sorghum linkage groups (Additional file 1: Table S1). These loci were selected based on their high polymorphism and compatibility for multiplexing. PCR followed the QIAGEN® multi-master mix kit protocol for SSR multiplex, and forward primers were labeled with different fluorescent dyes: FAM (6-carboxyfluorescein), HEX (hexachloro-6-carboxyfluorescein), or NED (2, 7, 8′-benzo-5′-fluoro-2′, 4,7-trichloro-5-carboxyfluorescein) (PE-Applied Biosystems, Foster City, CA). PCR was carried out in 12 μl total volume of reaction mix containing 1 μM of each primer pair in a multiplex, 1 μl of template DNA, 2.6 μl of sterile ddH2O, 6 μl of QIAGEN® Multiplex PCR 2X Master mix. Polymerase chain reactions were run in a Master cycler (Eppendorf™) with an initial denaturation step of 15 min at 95°C, followed by 35 cycles of 30 sec at 94°C, 90 sec at 58°C, 60 sec at 72°C, 30 minutes at 60°C, and held at 4°C following QIAGEN® protocol for microsatellite multiplexes.

To determine SSR fragment sizes, 2 μl of the PCR product was diluted with 14 μl of ddH2O and then 2 μl of the diluted PCR product was added to 14 μl of 36:1 Hi-Di-Formamide: GenScan™/350 Rox™ size standard in a 96 well microtiter plate and was denatured at 95°C for 5 minutes and cooled on ice for at least 5 minutes. Allele size scoring of the PCR fragments was done by ABI 3100 Genetic Analyzer (DNA sequencer) and sizes were read using the associated GeneMapper 3.7 software (Applied Biosystems Inc., CA, USA) and manually scored. To exclude the possible effects of imprecise DNA fragment sizes due to stuttering, large allele drop out, or null alleles on genotyping, the software Allelobin (Prasanth et al. 2006) was used to classify observed SSR allele sizes into representative discrete allele sizes using a variation of the least-square minimization algorithm of Idury and Cardon (1997).

### Data recording and statistical analysis

#### Quantitative phenotypic measurements

To estimate phenotypic diversity, data were measured from 160 cultivated *S. bicolor* individual plants (20 plants per site, which represents a population) in the field on seven common quantitative phenotypic traits following descriptors for sorghum (IBPGR/ICRISAT 1993). The measured traits were: head length (HDL) and width (HDW) (cm), flag leaf length (LL) and width (LW) (cm), total leaf number (LN) on main stalk, plant height (PLHT) (cm), and number of tillers (TIL). The quantitative phenotypic data were scaled to fit a normal distribution and subjected to simple descriptive statistics. Pearson’s coefficients of correlations were computed between all pairs of traits and their significance was tested using a t-test. Values from the correlation matrix were used to perform PCA using GenStat software.

#### SSR polymorphism and analysis of genetic diversity

To estimate the discriminatory power of the SSR loci, polymorphism information content (PIC) (Botstein et al. 1980; Anderson et al. 1993) was computed using PowerMarker software V3.25 (Liu and Muse 2005). The number and frequency of SSR alleles was also computed using the same software. GENEPOP 4.0 (Rousset 2008) was used to compute exact tests for Hardy-Weinberg equilibrium and for genotypic disequilibrium among pairs of loci. This was also complemented with the HW-QuickCheck computer program (Kalinowski 2006). Nei’s heterozygosity estimates (H_o_, H_s_, and H_t_) were computed using FSTAT software (Goudet 2002). Allelic richness (R_s_) and private allelic richness (R_p_) were computed using the rarefaction method (Hulbert 1971) implemented in HP-Rare 1.1 software (Kalinowski 2005). Significance of differences in the overall gene diversity, allelic richness and private allelic richness between populations and among the regions of collection was tested using a nonparametric Wilcoxon signed ranks test (Wilcoxon 1945) implemented in SPSS Statistics software release 17.

### Population structure and gene flow

To estimate the components of variance among regions of collection, and among and within populations, analysis of molecular variance (AMOVA) was computed using Arlequin v 3.1 software (Excoffier et al. 2005). To investigate population differentiation, Wright (1951) fixation index (F_ST_) of the total populations and pair wise F_ST_ among all-pairs of populations were computed using FSTAT software (Goudet 2002) and significance was tested based on 10000 bootstraps. Gene flow was estimated using indirect method based on the number of migrants per generation (N_m_) as (1-F_ST_)/4F_ST_. Shared alleles distance matrix (Jin and Chakraborty 1994) was used to construct Neighbor-joining dendrogram for the 160 samples belonging to the eight populations using PowerMarker (Liu and Muse 2005), and the resulting tree was viewed using TreeView 1.6.6 (Page 2001, available at http://taxonomy.zoology.gla.ac.uk/rod/rod.html). Further, the pattern of population structure and detection of admixture was visualized using a Bayesian model based clustering method implemented in STRUCTURE software, Version 2.2 (Pritchard et al. 2000). For this, two separate analyses were run with and without prior information about the populations. The first was done by assigning the site of collection as the putative population origin for each individual and the second run was without giving such information and letting the STRUCTURE program assign each individual into a population. The admixture model with correlated allele frequencies was used as suggested in the manual. A burn-in period of 10,000 was used followed by 10,000 Markov Chain Monte Carlo (MCMC) replications for data collection for K = 1 to K = 8 groups. For each K value, 10 replicates were run. This procedure clusters individuals into populations and estimates the proportion of membership in each population for each individual (Falush et al. 2003). The optimum number of clusters was predicted between K = 1 and K = 8 following the simulation method of Evanno et al. (2005) using the web based software STRUCTURE HARVESTER v0.6.92 (Earl and von Holdt 2012).

To study the pattern of gene flow, Slatkin’s F_ST_ matrix was first converted into Rousset (1997) genetic distance as F_ST_/(1-F_ST_) matrix. The geographic distance among the collection sites was computed from geographical coordinates marked with the aid of GPS using the web based Geographic Distance Matrix Generator (GDMG) version 1.2.3 software of the American Museum of Natural History, Center for Biodiversity and Conservation (http://biodiversityinformatics.amnh.org/open_source/gdmg/index.php). Later, the correlation between Rousset’s genetic distance matrix and the geographic distance matrix of the collection sites was calculated using the web based program IBDWS version 3.23 (available at http://ibdws.sdsu.edu/~ibdws/). Significance of the correlation was tested using Mantel (1967) test. Moreover, analysis of reduced major axis (RMA) regression (Hellberg 1994) was done to calculate intercept and slope of genetic and geographic distance matrices for inference of isolation by distance.

## Results

### Diversity of quantitative phenotypic traits

Considerable variation was observed among the populations for the measured quantitative phenotypic traits. The number of tillers was in the range of zero to five. Head length was as small as 11 cm and as large as 46 cm in some cultivars and head width was in the range of five to 40 cm with mean 12.1 cm. Plant height was in the range of 147 cm (in an improved lowland variety, 76T1#23, coded Wello-2) to 470 cm (Metekel-1) averaging 289 cm. Six of the eight populations showed an average height of greater than 3 m. Leaf width ranged from 4.4 cm (Wello-2) to 12.5 cm (Metekel-1). Leaf length was also in the range of 42 cm to 100 cm and leaf number was in the range of six (Wello-2) to 24 (Wello-4) (Table 2).

**Table 2:** **Simple descriptive statistics and principal component factor loadings of the measured quantitative phenotypic traits**

Population name	TIL	HDL	HDW	PLHT	LW	LL	LN
Gibe-1	0.6	31.5	17.3	324.0	9.5	77.1	17.0
Gibe-2	1.0	36.1	14.5	315.3	8.4	77.7	17.0
Metekel-1	0.6	29.6	12.4	359.4	8.1	75.6	16.0
Metekel-2	0.1	32.2	16.0	322.0	8.0	74.2	14.6
Wello-1	1.0	24.4	10.2	301.5	8.0	63.1	16.1
Wello-2	0.1	21.1	8.6	166.4	6.0	63.6	7.7
Wello-3	0.3	27.2	10.2	221.0	8.6	68.3	11.8
Wello-4	0.9	15.4	7.9	304.9	8.9	63.1	19.7
Total population							
Minimum	0	11	5	147	4.4	42	6
Maximum	5	46	40	470	12.5	100	24
Mean	0.6	27.2	12.1	289.3	8.2	70.3	15.0
±SE	0.073	0.590	0.421	5.619	0.115	0.834	0.311
Factor loadings							
PC1	−0.211	−0.285	−0.297	−0.445	−0.350	−0.277	−0.383
PC2	0.565	−0.295	−0.302	−0.014	−0.092	−0.362	0.211
PC3	0.348	0.384	0.172	−0.359	0.131	0.403	−0.381

**Figure 2:**
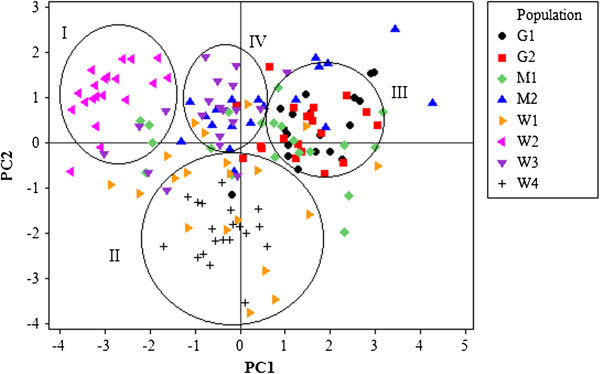
**PCA biplot showing the distribution of the 160 sorghum samples based on their measured phenotypes.**

Correlation was significant in all pairs of characters (p < 0.05), except between number of tillers and head length and width, and leaf length; between head length and leaf number, and between leaf length and leaf number. Plant height and leaf width had highly significant positive correlation with the remaining quantitative phenotypic traits. The first three principal component axes explained 80.53% of the total variation. Plant height contributed the largest factor loadings (0.44) for PCA1. PCA2 is mostly influenced by number of tillers per plant (0.56). For PCA3 leaf length contributed the largest share of the variation (0.40). Figure 2 shows the pattern of phenotypic diversity in the 160 plant samples from the eight populations based on the first two principal components. Four groups/ clusters are clearly observed in this biplot. Cluster I consisted of W2 populations. Cluster II composed of individuals from Wello populations, W1 and W4. Cluster III was dominated by Gibe populations, G1 and G2 with some individuals from M1 population with similar phenotypes. Cluster IV was mainly composed of individuals from W3. Metekel populations, M1 and M2 had individuals represented in all of the clusters.

### SSR polymorphism

Availability of alleles in each locus (the proportion of loci without missing alleles) ranged from 0.93 (Sb4-121) to 1.0 (Sb5-206, Sb1-1, Sb6-34, and Sb4-72) with mean 0.97. All of the 12 SSR loci were highly polymorphic with PIC values ranging from 0.38 to 0.85 (mean = 0.62) (Table 3). All except two SSR loci had PIC values greater than or equal to 0.5. They produced a total of 123 alleles of which 78 (63.4%) were rare (with frequency ≤ 0.05). The number of alleles produced per polymorphic locus ranged from 3 to 27 with an average of 10.25. The effective number of alleles was also in the range of 1.7 to 7.5 (Table 3). The frequency of the major allele was in the range 0.24-0.75 with a mean of 0.47. A comparison of SSR size ranges from the previously published reports and observed in the present study is presented in Additional file 1: Table S1. Tests for Hardy–Weinberg equilibrium (HWE) for all loci and all populations revealed that they did not significantly deviate from HWE.

**Table 3:** **Diversity indices of the SSR loci used in the study (N**_**A**_ **= observed number of alleles; A**_**e**_ **= effective number of alleles; R**_**s**_ **= Allelic richness; H**_**o**_ **= average observed heterozygosity; H**_**e**_ **= Expected heterozygosity/gene diversity; PIC = polymorphism information content;**

SSR loci	Availability	N_A_	A_e_	R_s_	H_o_	H_e_	PIC
Sb5-206	1.00	14	4.4	7.71	0.15	0.78	0.75
Sb1-1	1.00	27	7.5	10.00	0.15	0.87	0.85
Sb6-34	1.00	8	2.2	4.12	0.1	0.54	0.49
Sb5-256	0.94	3	2.5	3.00	0.33	0.60	0.53
Sb4-72	1.00	9	3.0	4.92	0.12	0.66	0.61
Sb6-84	0.99	12	3.9	6.93	0.12	0.75	0.73
Sb4-121	0.92	8	3.0	5.07	0.04	0.67	0.61
Sb6-342	0.94	10	3.9	5.35	0.09	0.75	0.69
Sb4-15	0.93	7	1.7	3.95	0.12	0.42	0.38
Sb5-236	0.96	9	3.1	5.28	0.17	0.68	0.63
Sb6-57	0.95	8	2.3	3.57	0.11	0.57	0.48
SBKAFGK1	0.97	8	3.2	3.80	0.07	0.69	0.62
Overall mean	0.97	10.25	3.4	5.31	0.13	0.66	0.61

### Genetic diversity

The values of the various genetic diversity indices of the eight populations are presented in Table 4. Average observed heterozygosity (H_o_) was in the range of 0.05-0.32 with mean 0.13 across all loci. Gene diversity was the lowest in Gibe-1 (H_e_ = 0.20) population and the highest in Wello-2 (H_e_ = 0.70) population and its value averaged over all populations and loci was 0.67 (SD = 0.11). W2 population had also the highest allelic richness. Allelic richness and private allelic richness over all pairs of populations and loci were significant (p < 0.05). M1 population had the highest (R_p_ = 0.83) and G1 had the lowest private allelic richness (R_p_ = 0.11). Wello as a region of collection supported the highest gene diversity (H_e_ = 0.70) whereas Gibe was the lowest (H_e_ = 0.40), but values were significant between Gibe and Wello (Z = −3.06, P = 0.002), and between Metekel and Wello (Z = −2.35, p = 0.01). Allelic richness was the lowest in Gibe (R_s_ = 3.9) and the highest in Wello (R_s_ = 6.8), but differences were significant between Gibe and Metekel (Z = −2.13, p = 0.03) and between Gibe and Wello (Z = −2.82, p = 0.006), but not significant between Metekel and Wello (Z = −1.78, p = 0.08). Similarly, private allelic richness was significant between Gibe and Metelkel (Z = −2.5, p = 0.01) and between Gibe and Wello (Z = −2.67, p = 0.008), but not significant between Metekel and Wello (Z = −0.71, p = 0.48).

**Table 4:** **Summary of the population diversity indices averaged over the 12 loci (N**_**A**_ **= number of alleles per polymorphic locus, A**_**p**_ **= number of private alleles, R**_**s**_ **= allelic richness, H**_**o**_ **= average observed heterozygosity, H**_**e**_ **= gene diversity)**

Population	N_A_	A_p_	R_s_	H_o_	H_e_
Gibe-1	2.1	1	1.88	0.10	0.13
Gibe-2	3.4	2	3.07	0.21	0.46
Metekel-1	4.6	12	3.84	0.12	0.42
Metekel-2	4.3	7	3.98	0.21	0.50
Wello-1	5.2	9	2.87	0.20	0.69
Wello-2	3.1	7	4.93	0.07	0.36
Wello-3	3.1	2	3.06	0.10	0.40
Wello-4	3.1	7	3.14	0.06	0.34

### Population genetic structure and gene flow

**Figure 3:**
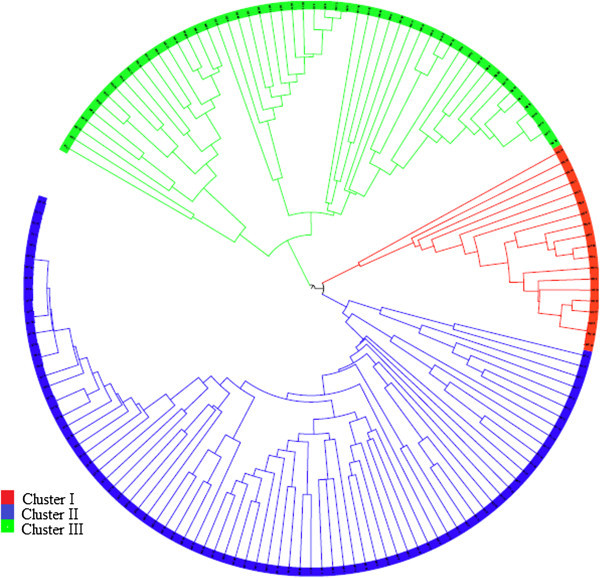
**Neighbor-joining radial tree showing the clustering pattern of individual samples from the eight sorghum landrace populations.**

**Figure 4:**
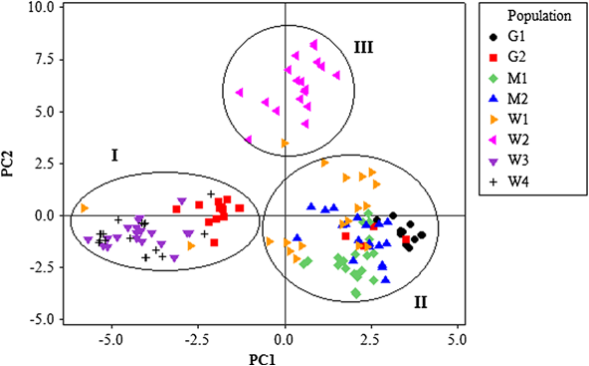
**Principal component (PCA) biplot of the 160 sorghum samples based on correlation of SSR allele frequencies.**

**Figure 5:**
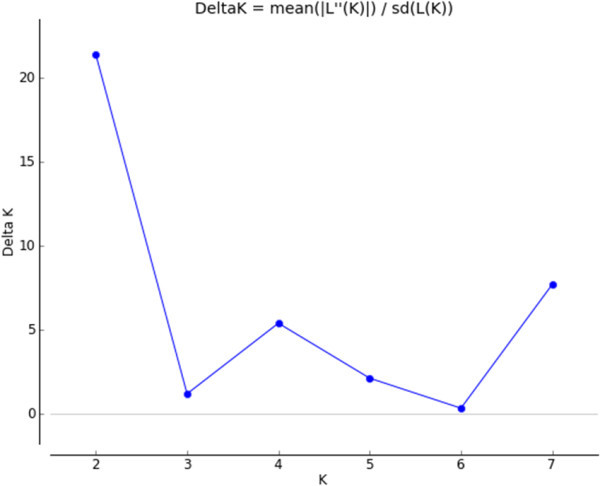
**A biplot detected the maximum peak at K = 2 (the optimum number of clusters) based on Evanno et al. (** 2005 **) prediction.**

**Figure 6:**
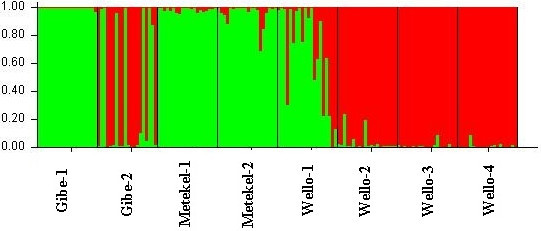
**STRUCTURE bar graphs of the 160 individual sorghum plant samples in eight pre-determined populations (x-axis) at K = 2.** Figures in the y-axis show coefficient of membership/assignment.

AMOVA showed 54.44% of the variation to be within populations, 32.76% among populations within regions, and 12.8% among the regions of origin (F_ST_ = 0.40, p < 0.001) (Table 5). Pair wise F_ST_ values among all populations were significant (p < 0.001) (Table 6). The divergence among the regions of collection was also high (F_ST_ = 0.21, p = 0.02). The Neighbor-joining dendrogram grouped the 160 individuals of the eight populations into three major clusters (Figure 3). Accordingly, Cluster I consisted of individuals from the improved early maturing variety, 76 T1#23 (Wello-2 population). Cluster II joined the two populations from Metekel (Metekel-1 and Metekel-2), a population from Wello (Wello-1) and Gibe-1 population. The third cluster (cluster III) consisted of individuals from the two adjacent Wello populations (Wello-3 and Wello-4) and Gibe-2 population. This pattern of clustering was also similar to the principal component biplot (Figure 4). Evanno et al. (2005) method on STRUCTURE outputs predicted K = 2 to be the most likely number of clusters (Figure 5). STRUCTURE with and without prior information on the populations gave similar clustering (K = 2). With no prior information, 73 (46%) of the total 160 individual plants were grouped in cluster I with ≥0.90 probability of membership whereas 71 (44%) of them were grouped in cluster II with the same probability of membership. Assigning the site of collection as the putative population origin for each individual (with prior information) resulted in exactly the same result as above (Figure 6). In such a case, both clusters contained 6 to 20 members of five populations each with ≥0.90 coefficient of ancestry. All of the 20 (100%) individuals of each of Metekel-1 and Gibe-1 populations, and 17 (85%) of individuals of Metekel-2 population were grouped in Cluster I (Additional file 2: Table S2). The number of migrants per generation as an indirect estimate of gene flow was very low (N_m_ = 0.38) in the overall populations. However, gene flow as high as N_m_ = 3.66 was observed in the adjacent Metekel populations (M1 and M2).

**Table 5:** **Analysis of molecular variance (AMOVA) among the sorghum regions of collection, among the populations within geographical regions, and within sorghum landrace populations**

Source of variation	d.f.	Sum of squares	Variance components	Percentage of variation	p value
Among geographical regions of collection	2	141	0.341	12.8	<0.001
Among populations within geographical regions	5	181.88	0.873	32.76	<0.001
Within populations	312	452.68	1.451	54.44	<0.001
Total	319	775.56	2.665

**Table 6:** **Pair wise F**_**ST**_ **matrix, a measure of population divergence among the sorghum landrace populations (all pairs were significant, p < 0.001)**

	G2	M1	M2	W1	W2	W3	W4
G1	0.396						
G2	0.440	0.324					
M1	0.327	0.223	0.061				
M2	0.350	0.201	0.176	0.100			
W1	0.651	0.431	0.500	0.405	0.308		
W2	0.692	0.430	0.471	0.430	0.295	0.547	
W3	0.694	0.345	0.484	0.445	0.357	0.565	0.389

**Figure 7:**
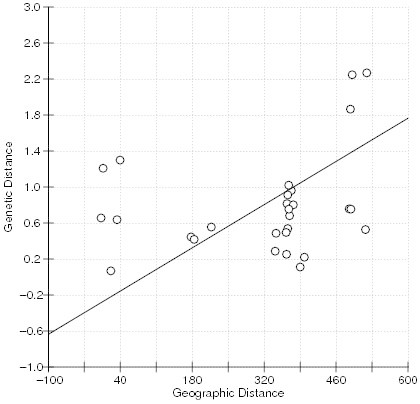
**RMA regression of Rousset’s genetic distance matrix plotted against the geographic distance (Km) matrix of the sorghum landrace collection sites in Ethiopia (** ***Y*** **= − 0.2936** ***X*** **+ 0.00343** **; R** ^**2**^ **= 0.074; p < 0.001).**

Mantel test for the correlation between Rousset’s genetic distance and the geographic distance matrices was weak, but significant (r = 0.272, p = 0.020). Moreover, the reduced major axis (RMA) regression showed a significant relationship with an intercept (−0.2936 ± SE0.2290, 1000 bootstraps over individual pairs), slope (0.003 ± SE0.0006) and with coefficient of determination (R^2^ = 0.074) (Figure 7).

## Discussion

### Diversity of quantitative phenotypic traits

It is well known that the majority of the Ethiopian sorghum landraces are characterized by high biomass (tall height, large leaf area and large number of leaves). All of the populations included in the present study displayed such characters except the only improved exotic variety, 76 T1#23 which showed parameters deviated from such measurements. For instance, all except Wello-2 and Wello-3 populations exhibited average height between 300 and 360 cm. Wello-3 (Jigurte) population is relatively shorter and earlier maturing than the high yielding and previously the dominant cultivar called Degalit (Wello-4). Jigurte is now becoming the dominant cultivar in Kobo-Alamata plain due to its earlier maturity than the other landraces and its better suitability to the changing climate, mainly to unreliable rainfall in the region. Some farmers call it as “America” perhaps because it was introduced from another place decades ago. The observed high variation in the range of the quantitative phenotypic traits in all populations could have genetic basis or it could be due to phenotypic plasticity. If it is due to the latter, it could be due to the differences in the rainfall and temperature as there was little variation in altitude of the collection sites (1088-1500 m) to bring about such changes. Ayana et al. (2000) studied the geographical pattern of quantitative phenotypic traits in Ethiopian and Eritrean sorghum gene bank accessions. They found that the variation within and among geographical regions was high and they suggested that gradients of growing period, rainfall and temperature are more important for such variations and should be considered during future germplasm collection.

### SSR polymorphism and genetic diversity

The observed SSR fragment sizes were within the range of the sizes in the previously published reports in sorghum (Brown et al. 1996; Dean et al. 1999; Ghebru et al. 2002; Agrama and Tuinstra 2003; Abu-Assar et al. 2005). These set of primer pairs are highly polymorphic and are being used for genetic finger printing as well as marker assisted breeding programs.

Although there was no comparison of the present *in situ* germplasm set with historical gene bank accessions, the high genetic diversity observed in Wello populations compared to the populations from other regions may indicate that the sorghum genetic diversity in this region is still in a good situation. This may show that farmers even during harsh drought seasons can conserve their landraces. However, this does not show the changes in the historical genetic diversity in the region. The highest diversity in Wello-2 population representing an exotic improved variety (76 T1#23) was rather unexpected. This variety was released in 1976 and its distribution to farmers all over the country has also long history. Thus, it might be because the variety was mixed with the landraces and lost its genetic purity. As a region of collection, Gibe populations were found to have the lowest diversity and Wello populations the highest in terms of allelic richness and number of private alleles. The significantly lower genetic diversity indices of the Gibe and Metekel populations than those of the Wello populations may indicate some level of genetic drift (but SSR analysis did not confirm this) during sampling of the seeds by migrants during settlement. Farmers usually carry few heads when they migrate and these may represent few genotypes only. Gibe and Metekel areas had no history of sorghum production before settlement.

The extent of the gene diversity of the studied Ethiopian sorghum populations (H_e_ = 0.66) was similar to Kenyan sorghum accessions (H_e_ = 0.66) (Ngugi and Onyango, 2012), Niger sorghum (H_e_ = 0.613) (Deu et al. (2008) and Eritrea sorghum (H_e_ = 0.65) (Ghebru et al. 2002), but higher than Morocco sorghum (H_e_ = 0.29) (Djé et al. 2000) and (H_e_ = 0.32) (Barnaud et al. 2007) using similar SSR markers. However, comparisons were not fair as the number of samples and the sampling strategy were different. Similarly, the observed heterozygosity (H_o_ = 0.13) was comparable to what was observed in Djé et al. (2000) (H_o_ = 0.134) and a bit higher than in Barnaud et al. (2007) (H_o_ = 0.11), but much higher than the result of Deu et al. (2008) (0.042). Although there was no significant departure from HWE, the observed heterozygosity was much lower than the expected hetrozygosity/gene diversity. In congruence with this study, Nybom (2004) compiled 79 microsatellite based studies and found that grand means for H_o_ was lower than H_e_ in 64 of these studies. Similarly, most of the genetic diversity studies in sorghum using SSRs (e.g., Ghebru et al. 2002; Barro-Kondombo et al. 2010; Deu et al. 2010; Ngugi and Onyango 2012) supported this finding.

Cuevas and Prom (2013) studied the genetic diversity and population structure of 137 sorghum accessions of Ethiopian origin preserved at USDA-ARS National Plant Germplasm System (NPGS) using 20 SSRs and found observed and expected heterozygosity of 0.23 and 0.78, respectively. These figures are higher than our findings. Even though *ex situ* accessions can sometimes experience loss of variability associated with missing of low frequency alleles (<1%) during repeated regeneration (e.g., Wilkes, 1989; Adugna et al. 2013), the major difference in the diversity of the present study and Cuevas and Prom (2013) was perhaps in the sampling strategy including the sampling area and period. Sorghum grows almost everywhere in Ethiopia between altitude range of 500 and 2400 m. However, our sampling mainly focused only on three geographical regions and individual plant sample collections were done in 3–5 farmers’ fields. Cuevas and Prom (2013) mentioned that most of the accessions they used had no passport data and thus there is a possibility that they could be countrywide collections. It is possible that including more locations may increase the chance of getting higher diversity.

### Population structure and gene flow

Some populations of landraces with different folk names like Degalit and Jigurte from Wello, which are morphologically different, were not found to be distinct using SSRs. This could be attributed to different reasons. First, they may not be genetically distinct from each other in which case the morphological differences between these cultivars may have little genetic basis; instead it could be due to farmers’ directional selection for different morphological traits for different purposes. Another reason could be that the observed morphological differences might not be detected using neutral genetic markers. Similar observations were made in Mali, Guinea and Kenya that varieties defined as different, based on their vernacular/ folk names or collection sites were in fact very closely related using SSR markers (Sagnard et al. 2011; Labeyrie et al. 2014). Collections were made in Metekel in settlement villages and Gibe valley composed mainly of people from Wello. Therefore, as expected, clustering together of Metekel-1 and Metekel-2 populations with Wello-1 and Gibe-1 populations might be due to long distance seed movement with settlers. Surprisingly, Gibe populations displayed more affiliation with Wello and Metekel populations than within themselves. High gene flow was also observed between Wello-1 and Metekel-2 (Nm = 2.25) and between Wello-1 and Metekel-1 (Nm = 1.14) populations as that of the gene flow between the adjacent Metekel-1 and Metekel-2 populations (Nm = 3.66). However, gene flow in the overall populations was very low, which was contradictory to the *ex situ* accessions of Ethiopian origin conserved at USDA-ARS (Cuevas and Prom 2013).

Mantel test for the correlation between Rousset’s genetic distance and the geographic distance matrices shows only the significance of the relationship; hence, slope and intercept of this relationship should be done using regression techniques (Bohonak 2002). Among the regression techniques, reduced major axis (RMA) is reportedly better for analysis of isolation by distance (Hellberg 1994). Hence, computation of intercept and slope of genetic distance of the sorghum landraces and the geographic distance of the collection sites using RMA regression resulted in a weak, but significant relationship with slope (0.003 ± SE0.0006). This shows that gene flow among populations follows a trend of isolation by distance (IBD) in a two dimensional stepping stone model, which indicates that the farthest the populations are located the weakest are their relationships. However, long distance seed movement as it has already happened from Wello area to Metekel by settlers could be the major force that played a major role in shaping the genetic structure of the landrace populations. Thus, we believe that the pattern of the population genetic structure of the studied landraces was strongly influenced by human migration with evidence from Figure 3. It is known that this pattern of genetic structure still observed today is the result of the history of domestication and human migrations (Sagnard et al. 2011).

### Implications for crop improvement and genetic resources conservation

The importance of crop diversity to counteract genetic vulnerability and how plant breeding, plant variety legislation, and an expanding seed industry may influence genetic diversity is well discussed elsewhere (e.g., Brown 1983). It has been argued that due to recurrent drought occurring in some of the major sorghum growing regions of the country, the diversity of the crop is declining over time and farmers in the dry lowlands tend to use high yielding improved early maturing sorghum varieties or shift their production systems to more vulnerable and low yielding early maturing crop species such as *tef* (*Eragrostis tef*), the dominant cereal, which might have resulted in genetic erosion of the sorghum landraces in these regions. The adoption of early maturing improved varieties was also found to be high in two of such areas affected by recurrent drought, Kobo in the North Eastern and Mieso in the Eastern parts of the country (Bekele et al. 2013). However, inadequate supply of seeds and lack of promotion impede the improved varieties to spread further in other regions of the country. Seed supply in the later regions is inadequate partly because the farmers decide to plant the seed of improved sorghum varieties late in the season only when the seed of their landraces fail to emerge. At this time, there is no possibility of getting improved seed except some kilos from the research institutes for some farmers for testing. During planting the improved seed, they usually do not remove the remnant plants of their landraces from the previous planting. As a result, the harvest from such fields does not ensure quality seed for the farmers for the next season planting. Due to this reason and lack of isolation from the neighboring fields, the improved seeds could not usually be used for more than two cycles. Another scenario for the lack of widespread adoption of improved varieties in the majority of the regions is the subsistence nature of poor sorghum farmers’ lives. They cannot afford to buy seeds of improved varieties. Because farmers harvest only from improved varieties, which are usually planted in small plots of land, no matter how much they love them, they consume what they have harvested. Thus, they will not get seed for the coming season and the problem persists. Unlike the improved seeds, seeds of the landraces can be shared freely, exchanged in kind or purchased from market at any time of the year.

In some areas like the extreme North Wello (where we collected Wello-2, 3, and 4 populations) once up on a time sorghum was the dominant crop and highly diverse. At present, however, *tef* is the dominant crop species in this area and wherever sorghum is growing, only few representative cultivars, which could go with the changing climate, are dominating. Shewayrga et al. (2012) proved loss of diversity in sorghum landraces in this region of Wello through comparing historical accessions preserved in gene banks for 30 years (originally collected in 1973) with *in situ* collections (newly collected in 2003).

The current Ethiopian sorghum germplasm holdings at the Ethiopian Institute Biodiversity (EIB) reached 9432 (http://www.ibc.gov.et/biodiversity/conservation/database-ms). This number is very small compared to the germplasm preserved elsewhere. For instance, more than 7000 germplasm accessions of Ethiopian origin are preserved at the US National Plant Germplasm System (Erpelding and Prom 2009) and another 4500 at ICRISAT genebank (IBC Institute of Biodiversity Conservation 2007). Moreover, even though the Ethiopian germplasm has been serving the world as source of valuable genes or for direct cultivation, the Ethiopian research system has not yet fully utilized these resources. As a result, there has been little success in breeding farmers’ preferred sorghum varieties in Ethiopia due to the mismatch between farmers’ preference and the breeders’ criteria for selection. Over the past 4 decades more than 40 varieties have been released for the different agroecologies except for the wet lowlands of Ethiopia including Metekel zone, which was covered by this study. However, none of the released varieties has been able to widely taken up by the farmers. Because the wet lowlands combine high moisture (humidity) and high temperature, they are suitable for the development of various fungal leaf and head diseases those attack sorghum. The breeding program has been almost exclusively dependent on introduced germplasm, which are short in height and early in maturity and little attention has been given to the landraces. However, there are landraces well adapted to the various sorghum growing environments due to co-evolution with the changing climate, insect pests, striga (the parasitic weed), and pathogens of the common diseases. Of course some of these landraces have limitations of poor grain quality and extended maturity of as long as nine months. On the other hand, better quality sorghum landraces are also found. Therefore, future sorghum improvement should focus on improving the landraces. For instance in the present study, crossing of the distinct Wello populations (Wello-3 and Wello-4) with some of the remaining populations included in this study may result in good combination for selection of progenies with desirable characteristics to be used as varieties in the wet lowlands as they are genetically distant from one another. Moreover, reintroduction of *ex situ* germplasm to their original places of collection which are now dispossessing the diversity may help to revitalize the lost diversity.

## Author’s information

AA has been a senior sorghum and millets breeder in the Ethiopian Institute of Agricultural Research (EIAR) for over 15 years. Currently, he is working for Advanta Seed International, a UPL Group company as a sorghum breeder for the African continent based in Ethiopia.

## Electronic supplementary material

Additional file 1: Table S1: Characteristics of the SSR primers (the repeat motifs, their chromosomal position, the size ranges and the optimum temperature) used in the study. (DOCX 14 KB)

Additional file 2: Table S2: Number of individuals from each pre-determined population assigned by STRUCTURE to the two clusters (k=1 and k=2) with ≥90% probability of membership. (DOCX 11 KB)

## References

[CR1] Abu-Assar AH, Uptmoor R, Abdelmula AA, Salih M, Ordon F, Friedt W (2005). Genetic variation in sorghum germplasm from Sudan, ICRISAT, and USA assessed by simple sequence repeats (SSRs). Crop Sci.

[CR2] Adugna A: *Population genetics and ecological studies in wild sorghum [Sorghum bicolor (L.)] in Ethiopia: implications for germplasm conservation*. Addis Ababa, Ethiopia: Addis Ababa University; 2012. [*PhD Thesis*]

[CR3] Adugna A, Snow AA, Sweeney PM, Bekele E, Mutegi E (2013). Population genetic structure of in situ wild Sorghum bicolor in its Ethiopian center of origin based on SSR markers. Genet Resour Crop Evol.

[CR4] Adugna A, Snow AA, Sweeney P (2011). Optimization of a high throughput, cost effective and all-stage DNA extraction protocol for sorghum. J Agric Sci Tech.

[CR5] Agrama HA, Tuinstra MR (2003). Phylogenetic diversity and relationships among sorghum accessions using SSRs and RAPDs. African J Biotech.

[CR6] Aldrich PR, Doebley J, Schertz KF, Stec A (1992). Patterns of allozyme variation in cultivated and wild Sorghum bicolor. Theor Appl Genet.

[CR7] Anderson JA, Churchill GA, Autrique JE, Tanksley SD, Sorrells ME (1993). Optimizing parental selection for genetic linkage maps. Genome.

[CR8] Appa RS, Prasada Rao KE, Mengesha MH, Reddy VG (1996). Morphological diversity in sorghum germplasm from India. Genet Resour Crop Evol.

[CR9] Ayana A, Bekele E (1998). Geographical patterns of morphological variation in sorghum (Sorghum bicolor (L.) Moench) germplasm from Ethiopia and Eritrea: qualitative characters. Hereditas.

[CR10] Ayana A, Bryngelsson T, Bekele E (2000). Genetic variation of Ethiopian and Eritrean sorghum (Sorghum bicolor (L.) Moench) germplasm assessed by random amplified polymorphic DNA (RAPD). Genet Resour Crop Evol.

[CR11] Ayana A, Bryngelsson T, Bekele E (2001). Geographic and altitudinal allozyme variation in sorghum (Sorghum bicolor(L.) Moench) landraces from Ethiopia and Eritrea. Hereditas.

[CR12] Barnaud A, Deu M, Garine E, McKey D, Joly HI (2007). Local genetic diversity of sorghum in a village in northern Cameroon: structure and dynamics of landraces. Theor Appl Genet.

[CR13] Barro-Kondombo C, Sagnard F, Chantereau J, Deu M, Vom Brocke K, Durand P, Goze E, Zongo JD (2010). Genetic structure among sorghum landraces as revealed by morphological variation and microsatellite markers in three agroclimatic regions of Burkina Faso. Theor Appl Genet.

[CR14] Bekele A, Sime M, Tirfessa A, Tesfaye E (2013). Socio-economic assessment of moisture stress sorghum growing areas of Mieso and Kobo districts. Research Report 98.

[CR15] Bhattramakki D, Dong J, Chhabra AK, Hart GE (2000). An integrated SSR and RFLP linkage map of Sorghum bicolor (L.) Moench. Genome.

[CR16] Bohonak AJ (2002). IBD (Isolation By Distance): a program for analyses of isolation by distance. J Heredity.

[CR17] Botstein D, White RL, Skolnick M, Davis RW (1980). Construction of a genetic linkage map in man using restriction fragment length polymorphisms. Amer J Human Genet.

[CR18] Brown WL (1983). Genetic diversity and genetic vulnerability – an appraisal. Econ Bot.

[CR19] Brown SM, Hopkins MS, Mitchell SE, Senior ML, Wang TY, Duncan RR, Gonzalez-Candelas F, Kresovich S (1996). Multiple methods for the identification of polymorphic simple sequence repeats (SSRs) in sorghum [Sorghum bicolor (L.) Moench]. Theor Appl Genet.

[CR20] Casa AM, Mitchell SE, Hamblin MT, Sun H, Bowers JE, Paterson AH, Aquadro CF, Kresovich S (2005). Diversity and selection in sorghum: simultaneous analyses using simple sequence repeats. Theor Appl Genet.

[CR21] (2011). Agricultural sample survey, report on area and production of major crops (private peasant holdings, Meher season), Statistical Bulletin, Vol I, September-December.

[CR22] (2012). Agricultural sample survey report on area and production of major crops (private peasant holdings, Meher season), Statistical Bulletin, Vol I, September-December.

[CR23] Cuevas HE, Prom LK (2013). Assessment of molecular diversity and population structure of the Ethiopian sorghum [Sorghum bicolor (L.) Moench] germplasm collection maintained by the USDA–ARS National Plant Germplasm System using SSR markers. Genet Resour Crop Evol.

[CR24] Cui YX, Xu GX, Magill CW, Schertz KF, Hart GE (1995). RFLP-based assay of Sorghum bicolor (L.) Moench genetic diversity. Theor Appl Genet.

[CR25] Dahlberg JA, Zhang X, Hart GE, Mullet JE (2002). Comparative assessment of variation among sorghum germplasm accessions using seed morphology and RAPD measurements. Crop Sci.

[CR26] Dean RE, Dahlberg JA, Hopkins MS, Mitchell SE, Kresovich S (1999). Genetic redundancy and diversity among ‘orange’ accessions in the U.S. national sorghum collection as assessed with simple sequence repeat (SSR) markers. Crop Sci.

[CR27] Deu M, Sagnard F, Chantereau J, Calatayud C, He´rault D, Mariac C, Pham JL, Vigouroux Y, Kapran I, Traore PS, Mamadou A, Gerard B, Ndjeunga J, Bezancon G (2008). Niger-wide assessment of *in situ* sorghum genetic diversity with microsatellite markers. Theor Appl Genet.

[CR28] Deu M, Sagnard F, Chantereau J, Calatayud C, Vigouroux Y, Pham JL, Mariac C, Kapran I, Mamadou A, Ge´rard B, Ndjeunga J, Bezanc¸on G (2010). Spatio-temporal dynamics of genetic diversity in Sorghum bicolor in Niger. Theor Appl Genet.

[CR29] Djé Y, Heuertz M, Lefebvre C, Vekemans X (2000). Assessment of genetic diversity within and among germplasm accessions in cultivated sorghum using microsatellite markers. Theor Appl Genet.

[CR30] Earl DA, von Holdt BM (2012). STRUCTURE HARVESTER: a website and program for visualizing STRUCTURE output and implementing the Evanno method. Conserv Genet Resour.

[CR31] Edwards KJ, Barker JHA, Daly A, Jones C, Karp A (1996). Microsatellite libraries enriched for several microsatellite sequences in plants. Biotechniques.

[CR32] Ejeta G, Grenier C, Gressel J (2005). Sorghum and its weedy hybrids. Crop ferality and volunteerism.

[CR33] Erpelding JE, Prom LK (2009). Response to anthracnose infection for a subset of Ethiopian sorghum germplasm. J Agric Univ Puerto Rico.

[CR34] Evanno S, Regnaut S, Goudet J (2005). Detecting the number of clusters of individuals using the software STRUCTURE: a simulation study. Mol Ecol.

[CR35] Excoffier L, Laval G, Schneider S (2005). Arlequin ver. 3.0: an integrated software package for population genetics data analysis. Evol Bioinfo Online.

[CR36] Falush D, Stephens M, Pritchard JK (2003). Inference of population structure using multilocus genotype data: Linked loci and correlated allele frequencies. Genetics.

[CR37] (2010). Crop data. FAO.

[CR38] Geleta M, Labuschagne MT, Viljoen CD (2006). Genetic diversity analysis in sorghum germplasm as estimated by AFLP, SSR and morpho-agronomical markers. Biodivers Conserv.

[CR39] Ghebru B, Schmidt RJ, Bennetzen JL (2002). Genetic diversity of Eritrean sorghum landraces assessed with simple sequence repeat (SSR) markers. Theor Appl Genet.

[CR40] Goudet J (2002). FSTAT, version 2.932.

[CR41] Gupta PK, Varshney RK, Sharma PC, Ramesh B (1999). Molecular markers and their applications in wheat breeding. Plant Breed.

[CR42] Hellberg ME (1994). Relationships between inferred levels of gene flow and geographic distance in a philopatric coral, Balanophyllia elegans. Evol.

[CR43] Hulbert SH (1971). The nonconcept of species diversity: a critique and alternative parameters. Ecology.

[CR44] (2007). Ethiopia: Second Country Report on the State of PGRFA to FAO, August 2007, Addis Ababa, Ethiopia.

[CR45] (1993). Descriptors for sorghum [Sorghum bicolor (L.) Moench]. International Board of Plant Genetic Resources, Rome, Italy.

[CR46] Idury RM, Cardon LR (1997). A simple method for automated allele binning in microsatellite markers. Genome Res.

[CR47] Jin L, Chakraborty R (1994). Estimation of genetic distance and coefficient of gene diversity from single-probe multilocus DNA fingerprinting data. Mol Biol Evol.

[CR48] Jordan DR, Tao YZ, Godwin ID, Henzel RG, Cooper M, McIntyre CL (1998). Loss of genetic diversity associated with selection for resistance to sorghum midge in Australian sorghum. Euphytica.

[CR49] Kalinowski S (2005). HP-RARE 10: a computer program for performing rarefaction on measures of allelic richness. Mol Ecol.

[CR50] Kalinowski ST (2006). HW-QUICKCHECK: an easy-to-use computer program for checking genotypes for agreement with Hardy–Weinberg expectations. Mol Ecol Notes.

[CR51] Labeyrie V, Deu M, Barnaud A, Calatayud C, Buiron M, Wambugu P, Manel S, Glaszmann JC, Leclerc C (2014). Influence of ethnolinguistic diversity on the sorghum genetic patterns in subsistence farming systems in Eastern Kenya. PLoS ONE.

[CR52] Li M, Yuyama N, Luo L, Hirata M, Cai H (2009). In silico mapping of 1758 new SSR markers developed from public genomic sequences for sorghum. Mol Breed.

[CR53] Liu K, Muse SV (2005). PowerMarker: integrated analysis environment for genetic marker data. Bioinformatics.

[CR54] Mace ES, Xia L, Jordan DR, Halloran K, Parh DK, Huttner E, Wenzl P, Kilian A (2008). DArT markers: diversity analyses and mapping in Sorghum bicolor. BMC Genomics.

[CR55] Mantel N (1967). The detection of disease clustering and a generalized regression approach. Cancer Res.

[CR56] Menkir A, Goldsbrough P, Ejeta G (1997). RAPD based assessment of genetic diversity in cultivated races of sorghum. Crop Sci.

[CR57] Menz MA, Klein RR, Unruh NC, Rooney WL, Klein PE, Mullet JE (2004). Genetic diversity of public inbreds of sorghum determined by mapped AFLP and SSR markers. Crop Sci.

[CR58] Morris GP, Ramu P, Deshpande SP, Hash CT, Shah T, Upadhyaya HD, Riera-Lizarazu O, Brown PJ, Acharya CB, Mitchell SE, Harriman J, Glaubitz JC, Buckler ES, Kresovich S (2013). Population genomic and genome-wide association studies of agroclimatic traits in sorghum. Proc Natl Acad Sci USA.

[CR59] Mutegi E, Sagnard F, Semagn K, Deu M, Muraya M, Kanyenji B, de Villiers S, Kiambi D, Herselman L, Labuschagne M (2011). Genetic structure and relationships within and between cultivated and wild sorghum (Sorghum bicolor (L.) Moench) in Kenya as revealed by microsatellite markers. Theor Appl Genet.

[CR60] Ngugi K, Onyango CM (2012). Analysis of the molecular diversity of Kenyan Sorghum Germplasm using microsatellites. J Crop Sci Biotech.

[CR61] Nkongolo KK, Nsapato L (2003). Genetic diversity in Sorghum bicolor (L.) Moench accessions from different ecogeographical regions in Malawi assessed with RAPDs. Genet Resour Crop Evol.

[CR62] Nybom H (2004). Comparison of different nuclear DNA markers for estimating intraspecific genetic diversity in plants. Mol Ecol.

[CR63] Page RDM (2001). TreeView 1.6.6.

[CR64] Prasanth V, Chandra S, Jayashree B, Hoisington D (2006). AlleloBin-a program for allele binning of microsatellite markers based on the algorithm of Idury and Cardon (1997)..

[CR65] Pritchard JK, Stephens M, Donnelly P (2000). Inference of population structure using multilocus genotype data. Genetics.

[CR66] Ramu P, Billot C, Rami J-F, Senthilvel S, Upadhyaya HD, Ananda Reddy L, Hash CT (2013). Assessment of genetic diversity in the sorghum reference set using EST-SSR markers. Theor Appl Genet.

[CR67] Reddy VP, Upadhyaya HD, Gowda CLL: **Current status of sorghum genetic resources at ICRISAT: their sharing and impacts.** *J SAT Agric Res* 2006.,**2**(1):

[CR68] Reddy BVS, Ramesh S, Reddy PS, Kumar AA (2009). Genetic enhancement for drought tolerance in sorghum. Plant Breed Rev.

[CR69] Ritter KB, McIntyre CL, Godwin ID, Jordan DR, Chapman SC (2007). An assessment of the genetic relationship between sweet and grain sorghums, within Sorghum bicolor ssp. Bicolor (L.) Moench, using AFLP markers. Euphytica.

[CR70] Rosenow DT, Quisenberry JE, Wedt CW, Clark LE (1983). Drought tolerant sorghum and cotton germplasm. Agric Water Manag.

[CR71] Rousset F (1997). Genetic differentiation and estimation of gene flow from F-statistics under isolation by distance. Genetics.

[CR72] Rousset F (2008). Genepop’007: a complete re-implementation of the GENEPOP software for Windows and Linux. Mol Ecol Resour.

[CR73] Sagnard F, Deu M, Dembe´le´ D, Leblois R, Toure´ L, Diakite´ M, Calatayud C, Vaksmann M, Bouchet S, Malle´ Y, Togola S, Traore PCS (2011). Genetic diversity, structure, gene flow and evolutionary relationships within the Sorghum bicolor wild–weedy–crop complex in a western African region. Theor Appl Genet.

[CR74] Schertz KF (1977). Registration of A2Tx2753 and BTx2753 sorghum germplasm. Crop Sci.

[CR75] Schlotterer C (2004). The evolution of molecular markers-just a matter of fashion?. Genetics.

[CR76] Shehzad T, Okuizumi H, Kawase M, Okuno K (2009). Development of SSR-based sorghum (Sorghum bicolor (L.) Moench) diversity research set of germplasm and its evaluation by morphological traits. Genet Resour Crop Evol.

[CR77] Shewayrga H, Jordan D, Godwin ID (2012). Genetic erosion and changes in distribution of sorghum (Sorghum bicolor L. Moench) landraces in north-eastern Ethiopia. Plant Genet Resour.

[CR78] Singh R, Axtell JD (1973). High lysine mutant gene (hl) that improves protein quality and biological value of grain sorghum. Crop Sci.

[CR79] Taramino G, Tingey S (1996). Simple sequence repeats for germplasm analysis and mapping in maize. Genome.

[CR80] Taramino G, Tarchini R, Ferrario S, Lee M, Pe ME (1997). Characterization and mapping of simple sequence repeats (SSRs) in Sorghum bicolor. Theor Appl Genet.

[CR81] Uptmoor R, Wenzel W, Friedt W, Donaldson G, Ayisi K, Ordon F (2003). Comparative analysis on the genetic relatedness of Sorghum bicolor accessions from Southern Africa by RAPDs, AFLPs and SSRs. Theor Appl Genet.

[CR82] (2010). Staple food value chain analysis.

[CR83] Van Beuningen LT, Busch RH (1997). Genetic diversity among North American spring wheat cultivars: III. Cluster analysis based on quantitative morphological traits. Crop Sci.

[CR84] Wang ML, Zhu C, Barkley NA, Chen Z, Erpelding JE, Murray SC, Tuinstra MR, Tesso T, Pederson GA, Yu J (2009). Genetic diversity and population structure analysis of accessions in the US historic sweet sorghum collection. Theor Appl Genet.

[CR85] Wang YH, Upadhyaya HD, Burrell AM, Sahraeian SME, Klein RR, Klein PE (2013). Genetic Structure and Linkage Disequilibrium in a Diverse, Representative Collection of the C4 Model Plant, *Sorghum bicolor*. G3.

[CR86] Wani SP, Albrizio R, Vaija NR (2011). Sorghum: Crop yield response to water.

[CR87] Wilcoxon F (1945). Individual comparison by ranking methods. Biometrics.

[CR88] Wilkes G, Knutson L, Stoner AK (1989). Germplasm preservation: objectives and needs. Biotic diversity and germplasm preservation, Global imperatives.

[CR89] Wright S (1951). The genetical structure of populations. Ann Eugen.

[CR90] Wu YQ, Huang Y, Tauer CG, Porter DR (2006). Genetic diversity of sorghum accessions resistant to greenbugs as assessed with AFLP markers. Genome.

[CR91] Yang W, de Oliveria AC, Godwin I, Schertz KF, Bennetzen JL (1996). Comparison of DNA marker technologies in characterizing plant genome diversity: variability in Chinese sorghums. Crop Sci.

[CR92] Zongo J, Gouyon PH, Sandmeier M (1993). Genetic variability among sorghum accessions from the Sahelian agroecological region of Burkina Faso. Biodiver Conserv.

