# Supplementary material for: Analysis of in situ diversity and population structure in Ethiopian cultivated Sorghum bicolor (L.) landraces using phenotypic traits and SSR markers
Source: Springerplus. 2014 Apr 30;3:212. doi: 10.1186/2193-1801-3-212 (PMC4033718; doi:10.1186/2193-1801-3-212)
Supplement: Supplementary file 1 — Additional file 1: Table S1: Characteristics of the SSR primers (the repeat motifs, their chromosomal position, the size ranges and the optimum temperature) used in the study. (DOCX 14 KB) [file 40064_2014_960_MOESM1_ESM.docx]

**Table S1** Characteristics of the SSR primers (the repeat motifs, their chromosomal position, the size ranges and the optimum temperature) used in the study

|  | Primer | Flanking sequences (5'-3') ^a^ | Repeat motif | CN^b^ | Size range (bp) | |  |
| --- | --- | --- | --- | --- | --- | --- | --- |
| Multiplex set |  |  |  |  | Earlier studies^c^ | This study | T_opt_^d^ |
| 1 | Sb5-206 | F: HEX-8ATTCATCATCCTCATCCTCGTAGAA  R: AAAAACCAACCCGACCCACTC | (AC)_13_/(AG)_20_ | 5 | 92-156 | 100-126 | 57 |
|  | Sb1-1 | F: FAM-6TCCTGTTTGACAAGCGCTTATA  R: AAACATCATACGAGCTCATCAATG | (AG)_16_ | 8 | 241-300 | 240-302 | 60 |
|  | Sb6-34 | F: HEX-8AACAGCAGTAATGCCACAC  R: TGACTTGGTAGAGAACTTGTCTTC | [(AC)/(CG)]_15_ | 9 | 168-208 | 171-205 | 60 |
|  | Sb5-256 | F: FAM-6AATTTGCTTTTTGGTCCGTTT  R: TAGGAAAGACAGTACTAGAGGTCA | (AG)_8_ | 3 | 162-214 | 165-171 | 58 |
|  | Sb4-72 | F: NED-TGCCACCACTCTGGAAAAGGCTA  R: CTGAGGACTGCCCCAAATGTAGG | (AG)_16_ | 2 | 182-350 | 184-210 | 60 |
| 2 | Sb6-84 | F: FAM-TAACGGACCACTAACAAATGATT  R: TAACGGACCACTAACAAATGATT | (AG)_14_ | 6 | 170-222 | 180-208 | 58 |
|  | Sb4-121 | F: NED-FAM-GAAAAATCTCCGTCAATCCCAAAAATAA  R: CGCTGAACAACGAAAGGAATAAGTG | (AC)_14_ | 4 | 200-229 | 214-228 | 60 |
|  | Sb6-342 | F: HEX-6TGCTTGTGAGAGTGCCTCCCT  R: GTGAACCTGCTGCTTTAGTCGATG | (AC)_25_ | 1 | 250-320 | 264-290 | 56 |
|  | Sb4-15 | F: HEX-GCTGCTAAGCCGTGCTGA  R: TTATTTGGGTGAAGTAGAGGTGAACA | (AG)_16_ | 5 | 119-135 | 118-136 | 57 |
|  | Sb5-236 | F: NED-6GCCAAGAGAAACACAAACAA  R: AGCAATGTATTTAGGCAACACA | (AG)_20_ | 7 | 158-222 | 168-184 | 57 |
|  | Sb6-57 | F: HEX-FAM-ACAGGGCTTTAGGGAAATCG  R: CCATCACCGTCGGCATCT | (AG)_18_ | 3 | 283-320 | 284-312 | 60 |
| 1 | SBKAFGK1 | F: FAM-6GCTTTCGGCGAGCATCTTACAA  R: GCGGTTGGATTCGCCATG | (AAC)_9_ | 10 | 140-320 | 239-278 | 60 |

^a^ (F= Forward primer, R= Reverse primer); ^b^CN = Chromosome number; ^c^ Earlier results were compiled from Brown et al. (1996), Dean et al. (1999), Ghebru et al. (2002), Agrama and Tuinstra (2003), Abu Assar et al. (2005); ^d^T_opt_= optimum temperature
