# Supplementary material for: Analysis of in situ diversity and population structure in Ethiopian cultivated Sorghum bicolor (L.) landraces using phenotypic traits and SSR markers
Source: Springerplus. 2014 Apr 30;3:212. doi: 10.1186/2193-1801-3-212 (PMC4033718; doi:10.1186/2193-1801-3-212)
Supplement: Supplementary file 2 — Additional file 2: Table S2: Number of individuals from each pre-determined population assigned by STRUCTURE to the two clusters (k=1 and k=2) with ≥90% probability of membership. (DOCX 11 KB) [file 40064_2014_960_MOESM2_ESM.docx]

**Supplementary Table 2** Number of individuals from each pre-determined population assigned by STRUCTURE to the two clusters (k=1 and k=2) with ≥90% probability of membership

| Population | K=1 | K=2 |
| --- | --- | --- |
| Gibe-1 | 20 | 0 |
| Gibe-2 | 6 | 12 |
| Metekel-1 | 20 | 0 |
| Metekel -2 | 17 | 0 |
| Wello-1 | 10 | 1 |
| Wello-2 | 0 | 18 |
| Wello-3 | 0 | 20 |
| Wello-4 | 0 | 20 |
